# Antibiotic Practice in Patients With Acute Abdomen Admitted to a Danish Regional Hospital: A Clinical Qualitative Assurance Study

**DOI:** 10.7759/cureus.37706

**Published:** 2023-04-17

**Authors:** Cihan Ozen, Ali Yalcinkaya, Kasra Zarandi, Ashraf Haydal, Sine Huus Pedersen, Peter Christian Leutscher

**Affiliations:** 1 General and Colorectal Surgery, Aalborg University Hospital, Aalborg, DNK; 2 Research and Development, Center for Clinical Research, Hjoerring, DNK; 3 Center for General Practice, Aalborg University Hospital, Aalborg, DNK; 4 General Surgery, North Denmark Regional Hospital, Hjoerring, DNK; 5 General Surgery, Aalborg University Hospital, Aalborg, DNK; 6 Pathology, North Denmark Regional Hospital, Hjoerring, DNK; 7 Research and Development, North Denmark Regional Hospital Centre for Clinical Research, Hjoerring, DNK; 8 Medicine, Aalborg University Hospital, Aalborg, DNK

**Keywords:** antibiotic resistance and behavioral compliance, antibiotic practices, antibiotic policies and guidelines, intra-abdominal infection, clostridium difficile, cephalosporins

## Abstract

Background

Acute abdomen is often complicated by intra-abdominal infection requiring antibiotic therapy. Danish regional antibiotic guidelines emphasize the restricted use of broad-spectrum antibiotics such as cephalosporins. In this study, we aimed to evaluate antibiotic practices in relation to hospitalized patients with acute abdomen.

Methodology

This retrospective quality assurance study was conducted among patients admitted to the surgical emergency department at the North Denmark Regional Hospital during a four-month observation period. Data were extracted from electronic patient journals and entered in the Research Electronic Data Capture data management system for further analytical work.

Results

Of 331 patients, 174 (53%) were treated with antibiotics, of whom 98 (56%) had been treated with cephalosporin, 47 (27%) with benzylpenicillin and gentamicin, 22 (13%) with piperacillin/tazobactam, and seven (4%) with ciprofloxacin. Use of a cephalosporin-based antibiotic regimen was significantly more common in patients with acute appendicitis (75%) compared to other diagnostic groups, such as acute cholecystitis (57%), incarcerated hernia with strangulation (56%), acute pancreatitis (50%), and acute diverticulitis (30%). However, patients with uncomplicated diverticulitis (53%) were significantly more often treated with benzylpenicillin and gentamicin, whereas patients with complicated diverticulitis Hinchey stage 3-4 were significantly more often treated with piperacillin/tazobactam. In addition, as the severity of acute cholecystitis increased, it was more frequently treated with piperacillin/tazobactam.

Conclusions

The study revealed that cephalosporins are frequently used in patients hospitalized with acute abdomen. This finding conflicts with current regional antibiotic guidelines. Reinforcement of the guidelines is required as an essential measure against the development of antibiotic resistance associated with the use of cephalosporins.

## Introduction

Acute abdomen is frequently associated with intra-abdominal infections (IAIs), such as acute appendicitis, diverticulitis, and cholecystitis, but may also be caused by other inflammatory conditions and non-specific abdominal pain in general. IAI is a broad term used for conditions in which inflammation of the peritoneum is observed due to local contamination with microorganisms, often followed by exudation in the abdominal cavity and a systemic inflammatory response [1].

Timely diagnosis and management of IAI by use of antibiotics and surgery as the key elements of intervention is an essential strategy [2,3]. Indication for use of antibiotics is thoroughly stated in the different clinical guidelines as part of the overall Danish rational antibiotic stewardship framework. Moreover, the criteria for the selection of antibiotics are also described in the guidelines aiming at a preferred use of narrow-spectrum antimicrobial agents rather than broad-spectrum agents, such as cephalosporins, quinolones, and carbapenems. Concern about increased bacterial resistance patterns, e.g., extended-spectrum beta-lactamases (ESBL), and more frequent occurrence of *Clostridium difficile* infections related to the use of broad-spectrum agents, in particular cephalosporins [4,5], are the main rationales for the preference of narrow-spectrum agents [6,7].

However, the use of antibiotics in the hospital sector in Denmark has increased by 25% in the last decade [7], which raises concern about the current rational antibiotic practice and use of broad-spectrum antibiotics. Epidemiological antibiotic data are systematically collected annually as part of a national antibiotic surveillance program in Denmark, but these data may not provide sufficient information about the underlying factors affecting antibiotic practice in the clinical setting. In this context, it is important to collect real-world data from the healthcare sector for current surveillance purposes.

Therefore, this study aimed to assess antibiotic practice with reference to the existing guidelines for patients with acute abdomen admitted to the surgical emergency department in a Danish regional hospital.

## Materials and methods

Study design and population

This quality assurance study was conducted at the North Denmark Regional Hospital to evaluate antibiotic therapy practice in the surgical emergency department. The target population comprised patients with acute abdomen admitted to the department during a four-month observation period. Patients were excluded from the study if they had received antibiotics within the previous four weeks before admission. In addition, patients diagnosed with gastrointestinal hemorrhage, trauma, small abscesses, and other medical diseases were also excluded.

Data collection and management

The Research Electronic Data Capture data management system was used in this study [8]. Demographic and clinical data were retrieved from patient medical files and included age, sex, body mass index (BMI), diagnosis using International Classification of Disease Tenth Edition (ICD-10), symptom duration before hospitalization, and rectal body temperature, in addition to other relevant data related to radiology examination findings, surgery performed, antibiotics administered, length of hospitalization, and pathology results. Quick Sequential (Sepsis-Related) Organ Failure Assessment (q-SOFA) scores were calculated [9].

Diagnostic categories of intra-abdominal infections

Groups for discharge diagnoses, including acute appendicitis, gallstones (with and without cholecystitis), diverticulitis, pancreatitis, incarcerated hernia, and non-specific abdominal pain, were created according to surgical findings together with radiological and pathological results. Moreover, subgroups within these groups were determined based on existing classification criteria. Acute appendicitis cases were categorized as either phlegmonous, gangrenous, or perforation/abscess based on the pathological examinations of resected specimens [10]. Diverticulitis was grouped into complicated and uncomplicated cases, and the Hinchey classification was used for the subgrouping of cases with complicated diverticulitis [11]. Likewise, cholecystitis in patients with gallstones was categorized as mild, moderate, or severe [12]. Pancreatitis severity was graded according to the Glasgow-Imrie criteria (mild and severe) [13]. Incarcerated hernias were categorized as strangulation verified and non-verified.

Antibiotics

Data on the antibiotic used in the perioperative periods were collected. Three principal antibiotic regimens were used according to the North Denmark regional antibiotic guidelines for patients admitted to the surgical emergency department. These included benzylpenicillin and gentamicin, cephalosporin (e.g., cefuroxime/ceftriaxone), and piperacillin/tazobactam. Moreover, quinolones (e.g., ciprofloxacin) were occasionally used. The listed antibiotics were often used in combination with metronidazole. Overall, antibiotic treatments were given only during their hospitalization.

Study registration

The study was registered in the North Denmark Region clinical research registry. Approval by the North Denmark Region Committee on Health Research Ethics was not deemed necessary as it is a clinical quality assurance study.

Statistical analysis

Nominal and ordinal parameters were described with frequency analysis. Means and standard deviations were used to describe scale parameters. Pearson’s chi-square and Fisher’s exact tests were used for differences between nominal and ordinal parameters. The Kolmogorov-Smirnov test was used for normality tests of scale parameters. The Mann-Whitney U and Kruskal-Wallis tests were used for differences between non-normally distributed scale parameters, whereas the independent-samples t-test was used for normally distributed scale parameters. Spearman’s rho correlation analysis was used for correlations between non-parametric variables. SPSS for Windows version 25.0 (IBM Corp., Armonk, NY, USA) was used with a 95% confidence interval and 0.05 significance level.

## Results

In total 331 patients with acute abdomen were included in this study. The mean age was 51 (SD = ±21) years and within the range of 8 to 95 years. Overall, 43% of the patients were males. The mean BMI of all patients was 27.4 (SD = ±6.1). The discharge diagnoses were as follows: non-specific abdominal pain (n = 95, 29%), acute appendicitis (n = 67, 20%), acute diverticulitis (n = 55, 16%), acute cholecystitis (n = 54, 16%), incarcerated hernia (n = 23, 7%), pancreatitis (n = 18, 6%), and other diagnoses (n = 19, 6%), including malign colon, sigmoid volvulus, small bowel ileus, perforated ulcer, and ischemic bowel (Table 1).

**Table 1:** Demographic and clinical characteristics in accordance with antibiotic therapy status. Other diagnoses included ileus, malign colon (acute), sigmoid volvulus, small bowel ileus, perforated ulcer, and ischemic bowel.

	Total	Antibiotic therapy		P-value
		Yes	No	
	N = 331	n = 174	n = 157	
Demography				
Age (years), mean (SD)	51 ± 21	54 ± 20	46 ± 21	<0.001
Sex, n (%)				
Male	142 (43)	76 (53)	66 (47)	0.425
Female	189 (57)	98 (52)	91 (48)	
Discharge diagnoses, n (%)				
Non-specific abdominal pain	95 (29)	4 (4)	91 (96)	<0.001
Acute appendicitis	67 (20)	67 (100)	0	<0.001
Phlegmonous	21 (31)	21 (100)	0	
Gangrenous	24 (36)	24 (100)	0	
Perforation/abscess	22 (33)	22 (100)	0	
Acute diverticulitis	55 (16)	33 (60)	22 (40)	<0.001
Uncomplicated	37 (67)	15 (41)	22 (59)	
Hinchey Stage 1-2	12 (22)	12 (100)	0	
Hinchey Stage 3-4	6 (11)	6 (100)	0	
Acute cholecystitis	54 (16)	35 (65)	19 (35)	<0.001
Mild - grade 1	20 (37)	3 (15)	17 (85)	
Moderate - grade 2	20 (37)	18 (90)	2 (10)	
Severe - grade 3	14 (26)	14 (100)	0	
Incarcerated hernia	23 (7)	16 (70)	7 (30)	0.222
Strangulation verified	11 (48)	9 (82)	2 (18)	
Strangulation non-verified	12 (52)	7 (58)	5 (42)	
Pancreatitis	18 (6)	8 (44)	10 (56)	0.412
Mild	15 (83)	6 (40)	9 (60)	
Severe	3 (17)	2 (67)	1 (33)	
Other diagnoses	19 (6)	11 (58)	8 (42)	0.287

Surgery was performed on 121 (37%) patients. Antibiotic treatment was given to 174 (53%) patients. The mean age of the antibiotic recipients was 54 (SD = ±20), which was significantly higher than the 46 years (SD = ±21) of the non-antibiotic recipients (p < 0.05) (Table 2).

**Table 2:** Demographic and clinical characteristics in accordance with selected antibiotic regimens. Other diagnoses included ileus, malign colon (acute), sigmoid volvulus, small bowel ileus, perforated ulcer, and ischemic bowel.

	Antibiotic regimen (N = 174)							P-value
	Benzylpenicillin and gentamicin		Cephalosporins		Piperacillin/tazobactam		Quinolones	
	n = 47		n = 98		n = 22		n = 7	
Demography								
Age (years), mean (SD)	53 ± 18		51 ± 21		71 ± 14		65 ± 10	<0.001
Gender, n (%)								
Male	19 (25)		46 (61)		10 (13)		1 (1)	0.471
Female	28 (29)		52 (53)		12 (12)		6 (6)	
Discharge diagnoses, n (%)								
Non-specific abdominal pain	-		1 (25)		3 (75)		-	<0.001
Apendicitis	12 (18)		50 (75)		4 (6)		1 (1)	<0.001
Phlegmonous	4 (19)		16 (76)		-		1 (5)	
Gangrenous	4 (17)		20 (83)		-		-	0.065
Perforation/abscess	4 (18)		14 (64)		4 (18)		-	
Diverticulitis	15 (46)		10 (30)		2 (6)		6 (18)	<0.001
Uncomplicated	8 (53)		3 (20)		-		4 (27)	<0.001
Hinchey stage 1-2	5 (42)		5 (42)		1 (8)		1 (8)	
Hinchey stage 3-4	2 (33)		2 (33)		1 (17)		1 (17)	
Cholecystitis	13 (37)		20 (57)		2 (6)		-	<0.001
Mild - grade 1	2 (67)		1 (33)		-		-	
Moderate - grade 2	7 (39)		11 (61)		-		-	<0.001
Severe - grade 3	4 (29)		8 (57)		2 (14)		-	
Incarcerated hernia	-		12 (75)		4 (25)		-	
Strangulation verified	-		5 (56)		4 (44)		-	
Strangulation non-verified	-		7 (100)		-		-	0.028
Pancreatitis	3 (37)		4 (50)		1 (13)		-	
Mild	2 (33)		3 (50)		1 (17)		-	0.705
Severe	1 (50)		1 (50)		-		-	
Other diagnoses	4 (36)		1(9)		6 (55)		-	<0.001
Surgery								
Yes	23 (19)		76 (63)		20 (16)		2 (2)	
No	24 (45)		22 (42)		2 (4)		5 (9)	<0.001

Antibiotic therapy of the different diagnostic groups

The distribution of antibiotic regimens in the 174 treated patients was as follows: penicillin and gentamicin (n = 47, 27%), cephalosporins (n = 98, 56%), piperacillin/tazobactam (n = 22, 13%), and quinolone (n = 7, 4%) (Table 2).

Antibiotic treatment was given to all 67 patients with appendicitis, and 75% of those had been treated with cephalosporins (Table 2). Among the 37 patients with uncomplicated diverticulitis, 15 (41%) had been treated with antibiotics. Patients with uncomplicated diverticulitis (53%) were significantly more often treated with benzylpenicillin and gentamicin. In addition, 27% of patients had been treated with quinolones, and 20% of patients had been treated with cephalosporins. All 18 patients with complicated diverticulitis received antibiotic therapy (Table 1). Hinchey stage 3-4 was significantly more often treated with piperacillin/tazobactam than uncomplicated diverticulitis (p < 0.05). Moreover, 39% of complicated diverticulitis Hinchey stage 1-4 patients were treated with cephalosporins (Table 2).

Antibiotics were used more frequently in patients with moderate/severe cholecystitis than in mild cholecystitis (p < 0.05) (Table 1). In the former group, 57% of patients were treated with cephalosporins (Table 2). Of these 34 patients, 53% were both operated on and received antibiotic treatment. On the other hand, 47% of these 34 patients had various comorbidities and were not operated on according to the clinical practices at that time and the results of the physicians’ own evaluations. Overall, 6% (n = 2) of moderate acute cholecystitis patients neither received antibiotic treatment nor underwent surgery.

Of the 23 patients with an incarcerated hernia, 30% did not receive antibiotic treatment, although it is recommended to initiate antibiotics in mesh repair hernia (Table 1). Moreover, among patients with confirmed strangulation, 56% were treated with cephalosporins (Table 2). Among the 15 patients with mild pancreatitis, six (40%) had been treated with antibiotics (Table 1). In addition, 50% of patients with pancreatitis taking antibiotics were treated with cephalosporins (Table 2).

## Discussion

In this study, cephalosporin was found to be the most prescribed antibiotic with an overall rate of 56%, as opposed to 27% for benzylpenicillin and gentamicin, although the latter regimen is the first-line antibiotic therapy in accordance with the regional antibiotic guidelines, especially in patients with acute appendicitis, acute diverticulitis, and acute cholecystitis. Preferably, cephalosporin should mainly be used in patients with penicillin allergy. In our study population, two patients only treated with cephalosporin had been registered with penicillin allergy.

Antibiotic treatment is generally recommended for the prevention of postoperative infectious complications [14]. In alignment with this approach, all patients with acute appendicitis in this study population were treated with antibiotics wherein cephalosporins were preferred in 75% of cases above the other available regimens. It is also recommended that patients suspected of acute complicated diverticulitis (abscess/perforation) should be treated with broad-spectrum antibiotic treatment early during clinical evaluation [15], whereas routine antibiotic therapy in patients with acute uncomplicated diverticulitis is not recommended, except for clinically complicated cases involving a weakened immune system, pregnancy, septicemia, and body temperature >38.5°C [16]. In our study population, 41% of uncomplicated diverticulitis patients had received antibiotic treatment due to the fulfillment of at least one of these criteria. Furthermore, for this group of patients with diverticulitis, a high proportion (27%) had been treated with quinolones, while an additional minor proportion (20%) had been treated with cephalosporins.

According to the Tokyo Guidelines 2018, in patients with moderate-to-severe acute cholecystitis, antibiotic therapy should be initiated in conjunction with cholecystectomy, whereas mild acute cholecystitis should not be treated with antibiotics [17]. In our study, 15% of patients with mild acute cholecystitis were treated with antibiotics. On the contrary, 6% of the patients with moderate-to-severe acute cholecystitis did not receive antibiotic treatment. In line with the other diagnostic groups, among 57% of the antibiotic-treated patients with acute cholecystitis, a cephalosporin agent was selected.

It is suggested that patients with incarcerated hernia operated on with a mesh repair should receive antibiotic treatment [18]. Among patients with confirmed hernia strangulation in this study, 56% were treated with cephalosporins, although piperacillin and tazobactam should be started according to the regional antibiotic guidelines.

Antibiotic treatment is not recommended in patients with acute mild pancreatitis [19]. About 40% of patients in this diagnostic group were treated with antibiotics, even if the treatment was in contradiction with regional antibiotic guidelines. However, antibiotic therapy is recommended in patients with acute severe pancreatitis confirmed by a CT scan [20]. The initial antibiotic choice according to our regional guidelines is piperacillin/tazobactam in patients with acute severe pancreatitis. Nevertheless, none of these patients received this antibiotic. In contrast, in this study, a cephalosporin agent was selected for 50% of acute pancreatitis patients. One of the three patients with acute severe pancreatitis did not receive antibiotics.

The use of cephalosporins is associated with an increased risk of antibiotic resistance development, which is a growing public health concern worldwide. Unjustified use of antibiotics and ineffective infection control policies are major causes of the spread of antibiotic resistance [21]. In accordance with the Danish Integrated Antimicrobial Resistance Monitoring and Research Program, the incidence of invasive infections is increasing in hospitalized patients in general but is of major concern in at-risk patient groups as well, such as the elderly and immunocompromised/chronically ill patients. In particular, the use of broad-spectrum antibiotics, such as cephalosporins, quinolones, and carbapenems, has been shown to contribute to the development of bacterial resistance, e.g., ESBL and carbapenemase-producing organisms. This is the reason why the Danish health authorities have developed a national antibiotic stewardship program aiming to limit the use of cephalosporins, quinolones, and carbapenems [22].

*C. difficile* infection is a common hospital-acquired infection and is an increasingly frequent cause of morbidity and mortality among the elderly and fragile hospitalized patients receiving broad-spectrum antibiotics, such as cephalosporins [23]. The development of *C. difficile* infection has been reported in 2.6% of surgical patients treated with cephalosporins. *C. difficile* emerges in the intestinal tract when the normal gut flora has been disrupted in conjunction with antibiotic therapy, in particular when using broad-spectrum agents, and is the causative organism of antibiotic-associated colitis including pseudomembranous colitis [24,25].

The study has revealed a considerable lack of compliance with the regional antibiotic treatment guidelines among clinicians prescribing antibiotics to patients. There seems to exist a preference toward second or third-generation cephalosporins over the benzylpenicillin and gentamicin antibiotic regimen, which is recommended as the first choice in the regional antibiotic guidelines. A plausible reason for this lack of compliance could be that clinicians are skeptical or cautious about the use of the benzylpenicillin and gentamicin antibiotic regimen due to concern about the risk of nephrotoxicity and ototoxicity associated with gentamicin administration and the risk of allergic reactions to penicillin. Another reason for the preference for cephalosporins could be that international antibiotic recommendations generally opt for this antibiotic because penicillin is not an option in many parts of the world due to widespread resistance. However, penicillin is still a solid option in Denmark as a result of many years of restrictive antibiotic treatment policy, thereby having prevented penicillin resistance in a successful manner. Moreover, the potential risk of nephrotoxicity and ototoxicity associated with gentamicin administration is carefully addressed in the regional antibiotic guidelines, e.g., by routine control of serum creatinine and plasma gentamicin concentrations.

There are some major limitations of this study. First, this was a single-center quality assurance study, which challenges the extent to which the findings can be generalized to other hospitals in Denmark. Second, the fact that it was a retrospective study limited the measurement and inclusion of other diagnoses that could be associated with the decisions for or against antibiotic therapy, or the use of different antibiotics. Finally, low patient numbers in certain diagnostic subgroups limited the potential outcomes of the statistical analyses.

## Conclusions

Our findings suggest a critically common use of cephalosporin in patients admitted to the hospital with acute abdomen. Hence, there is an urgent need for reinforcement of continuing medical training programs with a focus on an antibiotic stewardship strategy to increase adherence to the regional antibiotic guidelines. Such a strategy in Denmark aims to reduce the risk of antibiotic resistance development and adverse outcomes, such as *C. difficile* infection, in conjunction with the uncritical use of cephalosporins.
